# Current status and future perspective of external herbal dispensaries preparing traditional herbal medicine in South Korea: the first National-Wide Survey results

**DOI:** 10.1186/s12906-020-03094-0

**Published:** 2020-11-23

**Authors:** Soo-Hyun Sung, Ji-Eun Han, Ji-Yeon Ryu, Angela Dong-Min Sung, Jung-Youn Park, In-Hyuk Ha, Kyeong Han Kim, Jang-Kyung Park, Byung-Cheul Shin

**Affiliations:** 1 Department of Policy Development, National Development Institute of Korean Medicine, Seoul, 04554 Republic of Korea; 2 Department of Health and Welfare, Yuhan University, 590 Gyeongin-ro, Bucheon-si, Gyeonggi-do 14780 Republic of Korea; 3 Jaseng Hospital of Korean Medicine, 536 Gangnam-daero, Gangnam-gu Seoul, 06110 Republic of Korea; 4 Department of Preventive Medicine, College of Korean Medicine, Woosuk University, Wanju, 55338 Republic of Korea; 5 Division of Clinical Medicine, School of Korean Medicine, Pusan National University, Yangsan, Kyungnam 50612 Republic of Korea

**Keywords:** External herbal dispensary, Survey, Personalized herbal medicine, Traditional Korean medicine, Quality control, South Korea

## Abstract

**Background:**

An external herbal dispensary (EHD) is a type of pharmacy that provides various types of personalized herbal medicines (PHMs) to other traditional Korean medicine (TKM) institutions. Such dispensaries were legalized by the Ministry of Health and Welfare (MoHW) in 2008 in South Korea. The purpose of this study is to understand the current status of the EHD facilities and their quality controls and compare them with the good manufacture practice (GMP) guidelines to contribute to the establishment of the safety and quality control criteria for PHMs.

**Methods:**

We contacted 107 EHD representatives or people in charge of the preparation of PHMs (TKM pharmacists) and invited them to complete a survey questionnaire; of the total, 81 responded. The survey questionnaire was developed in 3 stages: drafting, revision by external experts, and final editing. It consisted of 20 questions covering 3 sections: basic characteristics of EHDs, facility, and quality control. The survey was hosted online from December 2017 to January 2018 as guided by the MoHW.

**Results:**

The completion rate was 75.7% (*n* = 81). In terms of facilities, the five facilities (water supply, manufacture, pest control, hygiene management and warehousing) that corresponded to the legal requirements of EHD were mostly equipped, but the types of facilities and equipment differed. Two facilities (sterilization and cross-contamination that were not legally required for EHD were found to have mostly pharmacopuncture-EHD (P-EHD), but hardly any herbal medicine-EHD (H-EHD). In our findings regarding quality control of non-medicinal herbs, sensory evaluation that included checks for foreign bodies and deterioration were conducted. In terms of the quality control of herbal medicines, residual pesticides and heavy metals tests were performed and for pharmacopuncture, pH, salinity, sterility, and endotoxin tests along with gross examination were performed. In the end, we found that 6 of the 38 standard items as required by the Korea GMP were suitable.

**Conclusions:**

In this study, detailed information for each existing EHD law was determined through a nationwide questionnaire. Moreover, the basis for its reflection in additional legal standards should be introduced so that safe herbal medicine can be prepared in EHDs.

## Background

With the increasing use of traditional medicine (TM), including herbal medicines (HMs), the safety and efficacy of the use of TM has also become important [1]. TM in East-Asia is known to have originated in China almost 3000 years ago [2]. It was introduced to South Korea around the tenth century and subsequently, led to the establishment of traditional Korean medicine (TKM) with its own unique characteristics [3]. Despite the introduction of conventional medicine in the nineteenth century, TKM remains a key part of the Korean medical system [3, 4]. Based on the 2017 National Survey for the usage of TKM and consumption of HMs, 73.8% of Koreans had experience using TKM [5]. The 2014 prevalence rate of the use of TKM treatments were 91.2% for acupuncture, 38.2% for HMs, 30.8% for cupping therapy, and 27.6% for moxibustion [6].

HMs are typically regulated by the Ministry of Food and Drug Safety (MFDS) of each country and manufactured by pharmaceutical companies with good manufacture practice (GMP) processes and facilities [7]. However, in some countries where TM is practiced such as South Korea, China and Japan, the TM institutions, such as clinics or hospitals, prepare personalized herbal medicines (PHMs) by mixing and processing medicinal herbs [8–10]. These PHMs are self-prepared in TKM clinics, and account for 39.0 to 52.8% of all HMs prescribed by TKM institutions in South Korea [5]. Further, PHMs are different from the ready-made products of pharmaceutical companies, as they are based on personalized prescriptions that correspond to the traits and symptoms of patients [10]. However, currently, there is a need to develop a set of proper management criteria in order to ensure that its safety and quality is at a similar level to that of ready-made medicines [11].

In the case of South Korea, PHMs are prepared in external herbal dispensaries (EHDs) or in TKM institutions such as clinics or hospitals [5]. An EHD is a type of pharmacy that provides various types of PHMs to other TKM institutions in South Korea. Such dispensaries were legalized by the Ministry of Health and Welfare (MoHW) in 2008. Generally, the EHD is established outside of a TKM institution and concoct PHMs or prepare medicinal acupunctures (pharmacopunctures) in accordance based on prescriptions from doctors in TKM institutions with whom they have a contractual relationship [12]. TKM clinics, in most cases, prepare and concoct a small amount of PHMs (decoction type), while EHD may prepare pills, liquid, tablets, capsules, and pharmacopunctures despite limitations of resources and quality control issues [13, 14]. There are 2 types of EHDs. An herbal medicine-EHD (H-EHD) prepares herbal medicines such as decoction type medicines, pills, tablets, liquid, capsules, or other oral medicines, while a pharmacopuncture-EHD (P-EHD) prepares pharmacopunctures such as sterile injection medicines to be injected at acupunctural or meridian points [15]. The 2 EHDs are subject to the same control criteria according to the Medical Act, in which the criteria for facilities are also specified. However, there are no quality control criteria for PHMs [15].

South Korea is a member state of the Pharmaceutical Inspection Co-operation Scheme (PIC/S), and a TKM clinic is classified as a medical institution. Therefore, they are required to follow the GMP, good laboratory practice (GLP) and good clinical practice (GCP) guidelines [16, 17]. These apply to the safety, efficacy, and quality management of the HMs manufactured by pharmaceutical companies at all times. However, the PHMs prepared by the herbal dispensaries of a TM institution or an EHD can be used if the seven criteria including the five standards for facilities and the one for manpower and hygienic management in the Medical Act are met [18]. This also applies to the preparation of pharmacopunctures. Despite this, there had been no separate quality control criteria until now [19].

Moreover, TKM institutions have to use medicinal herbs manufactured by the herb-GMP facilities that are licensed by the MFDS [20]. The amount of hazardous substances (e.g., heavy metals, pesticides, aflatoxins, sulfur dioxide, and benzopyrene) in medicinal herbs is restricted by the Regulations on Limits and Test Methods for Residues and Contaminants in Herbal Medicines [21]. However, when non-medicinal herbs other than the 601 types managed by the MFDS are used [22] or if herbal medicine is made in an inferior facility, it is difficult to predict what hazardous situations patients can be faced with upon taking HM.

This is the first government-supported study that investigates the current status of facilities and quality control in EHDs where PHMs are prepared in South Korea. This study aims to examine the current status of EHD and compare it to the GMP guidelines to ensure that there are improvements regarding safe use and quality control of PHMs. Further, these findings will contribute to the development of safety management criteria for PHMs in the countries where traditional medicine is practiced.

## Methods

### Study design

A cross-sectional study design has been used to examine the current status of EHD.

### Study sample

The study samples were composed of the 107 EHDs (H-EHD: 91, P-EHD: 16) that were registered with the Community Health Center of South Korea.

### Questionnaire development for initial draft

The questionnaire was designed in a 3-step process: initial drafting, revision based on expert opinion, and final editing. Six TKM experts participated in drafting the questionnaire (including 3 TKM specialists with an average of 10+ years of clinical experience, 2 PhD holders in TKM with 10+ years of experience in the field, and 1 TKM policy researcher who is also an accreditor of EHD of TKM institutions). The questionnaire was developed according to previous studies [13, 23–25]. The draft questionnaire was reviewed by 2 TKM researchers. Based on the reviewers’ comments, we made the following changes: regarding the basic status of EHD installation year, the EHD system has been implemented since 2009, so it was made into an objective format from 2009 to 2017 to obtain accurate information; regarding the facility status, the five facilities (facility of water supply, manufacture, pest control facility, hygiene management facility and warehousing) that meet the EHD legal requirements are essential. After reviewing the first draft, the research team met to consider the reviewers’ comments on each item in the questionnaire and to revise or maintain the items. For the items where no consent was reached, the 2 reviewers discussed these points with each other and made decisions based on consensus.

The questionnaire was designed to address the 2 types of EHDs: H-EHD and P-EHD. Participants could answer with either “Yes” or “No” for each question regarding the equipment and facilities in the EHDs and the current status of their quality control. Additionally, if there were no answers corresponding to the facilities or quality control methods in place, the participants were allowed to describe their answers in free form, to ensure that there were no missing data. The developed survey questionnaire was based on basic status (7 items), facility status (9 items), and quality control status (4 items) with a total of 20 survey items.

### Questionnaire development for second draft

The second draft of the questionnaire was revised based on the opinions of external experts who had a broader field of expertise. The finalized first draft was sent to 6 external experts from a variety of disciplines via e-mail to gather their comments. The external experts were a professor of TKM university who is a pharmaceutical board member at the national TM hospital, a TKM professor who majored in HMs, an evaluator from the Korea Agency of Hazard Analysis and Critical Control Point Accreditation and Services (KAHAS, a food safety and hygiene certification body and a public institution under the MFDS), a general manager of an EHD with 8+ years of experience in preparing pharmacopunctures, a TKM pharmacist working in an EHD with 8+ years of experience in preparing PHMs, and a quality control (QC) team leader from a pharmaceutical company that manufactures herbal drugs. The review opinions from the experts were as follows: the liquid for the pharmacopunctures made by the EHDs is a sterile preparation that is injected into the muscle or skin and is managed at a higher level than that of oral HM, so the detailed items on the facilities and the quality control questionnaire should be composed based on P-EHD.

Based on the opinions and comments from the second panel of experts, the research team had a further discussion to finalize the survey questionnaire. The finalized survey questionnaire, after the first and second rounds of reviewing, contained three categories surveying basic, facility, and quality control statuses, with a total of 20 survey items (Table 1) (See Additional File 1 for the final version of the questionnaire).

**Table 1:** Survey categories and items of questionnaire

Survey Categories (Survey Item)	Items	Survey Items
**I. Basic status**	**5**	1. Types of external herbal dispensary 2. Location 3. Opening year 4. Total area 5. Type of medical institution
**II. Facility status**	**11**	1. Self-quality inspection facility 2. Hygiene management facilities 3. Pest control facilities 4. Extraction, evaporation, or distillation equipments 5. Sterilization facilities 6. Filling facilities 7. Foreign body inspection facilities 8. Warehouse of raw materials, semi-finished, or final products 9. Cleanliness management facilities 10. Cross-contamination prevention facilities 11. Water supply facilities
**III. Quality control status**	**4**	1. Frequency of monitoring the temperature and humidity 2. Management of poisonous herbs 3. Quality control of non-medicinal herbs 4. Quality control of final products
**Total**	**20**	

### Questionnaire distribution

The survey questionnaire was hosted online, and was conducted from December 2017 to January 2018. The researchers secured the list of all 107 EHDs (H-EHD: 91 and P-EHD: 16), which were registered with the community health centers with assistance from the MoHW. In particular, the government emphasized that the survey was for designing policies regarding EHDs.

The survey was conducted by a specialized survey company called Research Korea (http://www.researchk.com). Employees of Research Korea contacted every EHDs with the contact information obtained. They then explained the aims, questionnaire development procedure and survey method, and also that personal information would be protected by statistical law. The questionnaire was then sent via e-mail only to those (107) who gave their consent.

In order to increase the participation rate of EHDs, we also utilized various methods such as announcing the importance of the survey to improve the policy on the development of EHDs by presenting official documents of cooperation with the MoHW, and providing small gifts of gratitude. The questionnaires were completed by the officers who were in charge of the preparation of PHMs in each EHD (TKM pharmacist) or the representing officers of the TM institutions with a dispensary so as to depict the current status as accurately as possible.

### Statistical analysis

The data from the H-EHDs and the P-EHDs were separately analyzed by an independent statistician. First, 70 H-EHD and 11 P-EHD samples were gathered, and an explore data analysis (EDA) was conducted. All data was validated and checked by the research team to ensure completeness. Where data was missing or incomplete, the Research Korea assistants followed up with the respondents through phone or email. The final data were coded, edited and statistically analyzed through descriptive analysis using SPSS version 21.0. (IBM, Armonk, NY, USA). A summary of the data was prepared, including the average, standard deviations, and frequency (n, %).

### Comparison of drug management of Korea GMP (KGMP), EHD, and survey items

Data on KGMP were collected based on MFDS guidance on GMP for medicinal products [26] and the legal requirements of EHD data from MoHW’s Medical Act [18]. The researchers compared and matched the legal requirements of EHDs and the survey items of EHDs based on the detailed items for each KGMP category. Disagreements between researchers were resolved by discussion.

## Results

### Completion rate of survey

For the survey, the questionnaires were distributed to the 107 EHDs that gave their consent in advance. A total of 81 EHDs (completion rate 75.7%) returned the questionnaires after completing them. Of the 91 H-EHDs, 70 (completion rate 76.9%) responded, while 11 out of 16 P-EHDs did so (68.8%).

### Basic characteristics of external herbal dispensaries

Table 2 shows the characteristics of EHD which participated in this survey. Out of the 11 P-EHDs, 4 P-EHDs (36.4%) were located in the Incheon/Gyeonggi province, and 3 P-EHDs (27.3%) were in the Pusan/Gyeongnam province. Regarding H-EHDs, 27 H-EHDs (38.6%) were located in the Seoul, and 25 H-EHDs (35.7%) in the Incheon/Gyeonggi province. All EHDs have been opened since 2009. Regarding the floor areas of EHD, 4 P-EHDs (36.4%) were between 165 and 330 m^2^ in size, and 22 H-EHDs (31.4%) were under 165 m^2^. Most P-EHDs (81.8%) and H-EHDs (65.7%) were opened by TKM clinics rather than by TKM hospitals.

**Table 2:** Demographics of EHDs and types of medical institution that open EHD

Characteristics	P-EHD	H-EHD
**No. of EHD**	**11 (100.0)**	**70 (100.0)**
TKM clinics	9 (81.8)	46 (65.7)
Network TKM clinics	2 (18.2)	17 (24.3)
TKM hospital	–	3 (4.3)
Public health center	–	2 (2.9)
General hospital	–	2 (2.9)
**Location**		
Seoul	1 (9.1)	27 (38.6)
Incheon/Gyeonggi province	4 (36.4)	25 (35.7)
Daejeon/Sejong/Chungcheong province	–	1 (1.4)
Gangwon province	1 (9.1)	–
Gwangju/Jeolla province	1 (9.1)	1 (1.4)
Daegu/Gyeongbuk province	1 (9.1)	2 (2.9)
Pusan/Gyeongnam province	3 (27.3)	14 (20.0)
**Opening year**		
2009	–	13 (18.6)
2010–2012	1 (9.1)	18 (25.7)
2013–2015	5 (45.5)	21 (30.0)
2016–2017	5 (45.5)	18 (25.7)
**Area of EHD (m**^**2**^**)**		
< 165	3 (27.3)	22 (31.4)
165–330	4 (36.4)	19 (27.1)
330–660	1 (9.1)	14 (20.0)
≥ 660	3 (27.3)	15 (21.4)

*EHD* external herbal dispensary, *P-EHD* pharmacopuncture-EHD, *H-EHD* herbal medicine-EHD

### Facility status of pharmacopuncture-external herbal dispensaries (P-EHD)

90.9% (*n* = 10) of P-EHDs had a self-quality inspection facility, while 9.1% (*n* = 1) referred the inspection to an accredited institution.

**Fig. 1:**
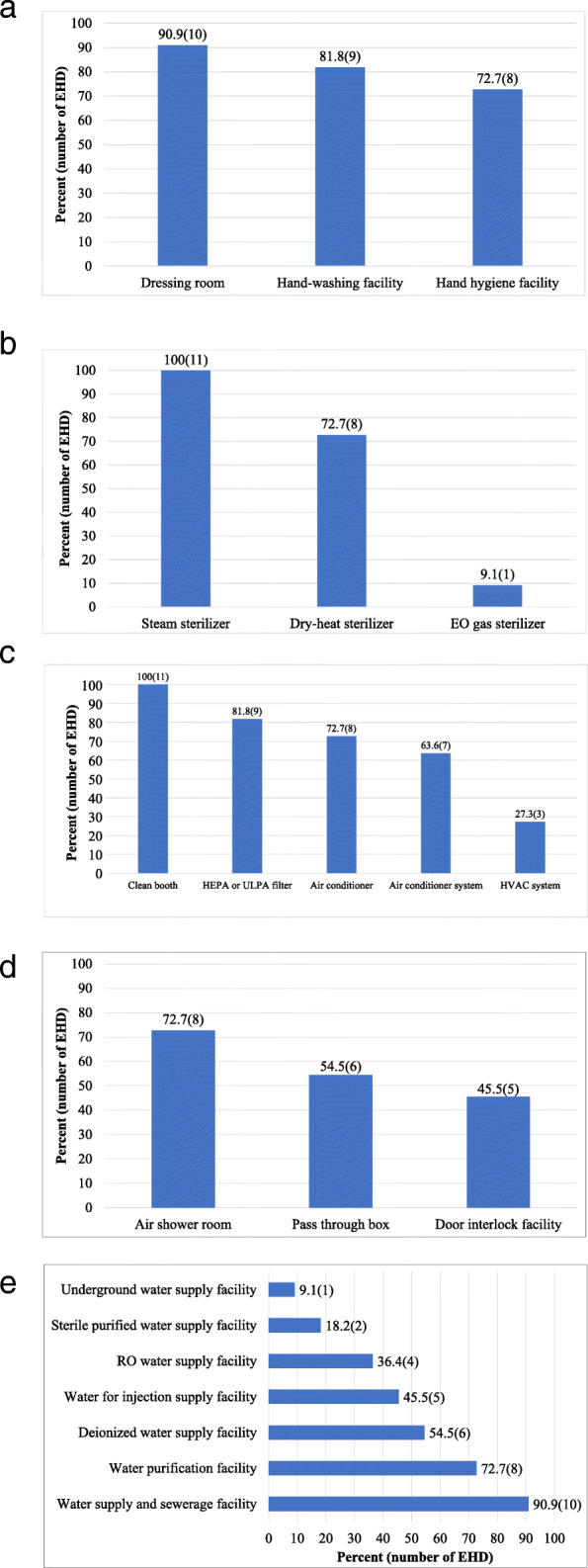
Facility status in pharmacopuncture-external herbal dispensary (P-EHD). EO: Ethylene Oxide; HEPA: High Efficiency Particulate Air; HVAC: Heating, Ventilation, & Air Conditioning; RO: Reverse Osmosis; ULPA: Ultra-Low Particulate Air

Regarding hygiene management facility, 90.9% (*n* = 10) of P-EHDs had dressing rooms, while 81.8% (*n* = 9) had a hand-washing room. 72.7% (*n* = 8) had a hand disinfection facility (Fig. 1(a)).

Regarding pest control facility, 45.5% (*n* = 5) of P-EHD had pest control lamps, 27.3% (*n* = 3) had ultrasonic devices, and 27.3% (*n* = 3) had pest traps.

81.8% (*n* = 9) of the P-EHDs had extraction equipment, 72.7% (*n* = 8) a distiller, and 63.6% (*n* = 7) an evaporator

With regards to sterilization facilities, 100.0% (*n* = 11) of P-EHDs had a steam sterilizer, 72.7% (*n* = 8) a dry-heat sterilizer, and 9.1% (*n* = 1) an ethylene oxide (EO) gas sterilizer (Fig. 1(b)).

81.8% (*n* = 9) of P-EHDs had semi-automatic filling equipment, 45.5% (*n* = 5) had manual filling equipment, and 36.4% (*n* = 4) had automatic filling equipment.

45.5% (*n* = 5) of the P-EHDs had a manual foreign body inspection facility, 27.3% (*n* = 3) had a semi-automatic foreign body inspection facility, and 9.1% (*n* = 1) had an automatic foreign body inspection facility.

81.9% (*n* = 9) of the P-EHDs had a raw material warehouse and final product warehouse, while 27.3% (*n* = 3) had a semi-finished product warehouse.

With regard to their cleanliness management facilities, all P-EHDs had a clean booth (100.0%), 81.8% (*n* = 9) had high efficiency particulate air (HEPA) or ultra-low particulate air (ULPA) filters, 72.7% (*n* = 8) had air conditioners, 63.6% (*n* = 7) had air conditioner systems, and 27.3% (*n* = 3) had heating, ventilation, & air conditioning (HVAC) systems (Fig. 1(c)).

Regarding cross-contamination prevention facilities, 72.7% (*n* = 8) of P-EHDs had an air shower room, while 54.5% (*n* = 6) had a pass-through box and 45.5% (*n* = 5) a door interlock facility (Fig. 1(d)).

Concerning their water supply facilities, 90.9% (*n* = 10) of P-EHDs had a water supply and sewerage facility, 72.7% (*n* = 8) had water purification facility, 54.5% (n = 6) had deionized water supply facility, 45.5% (n = 5) had water for injection supply facility, 36.4% (*n* = 4) had reverse osmosis (RO) water facility, 18.2% (*n* = 2) had sterile purified water supply facility, and 9.1% (*n* = 1) underground water supply facility (Fig. 1(e)).

### Facility status of herbal medicine-external herbal dispensaries (H-EHD)

7.1% (*n* = 5) of H-EHDs had a self-quality inspection facility. 25.7% (*n* = 18) referred the inspection to an accredited institution. 30.0% (*n* = 21) referred their inspections to pharmaceutical companies, and 37.2% (*n* = 26) did not performed any inspection.

**Fig. 2:**
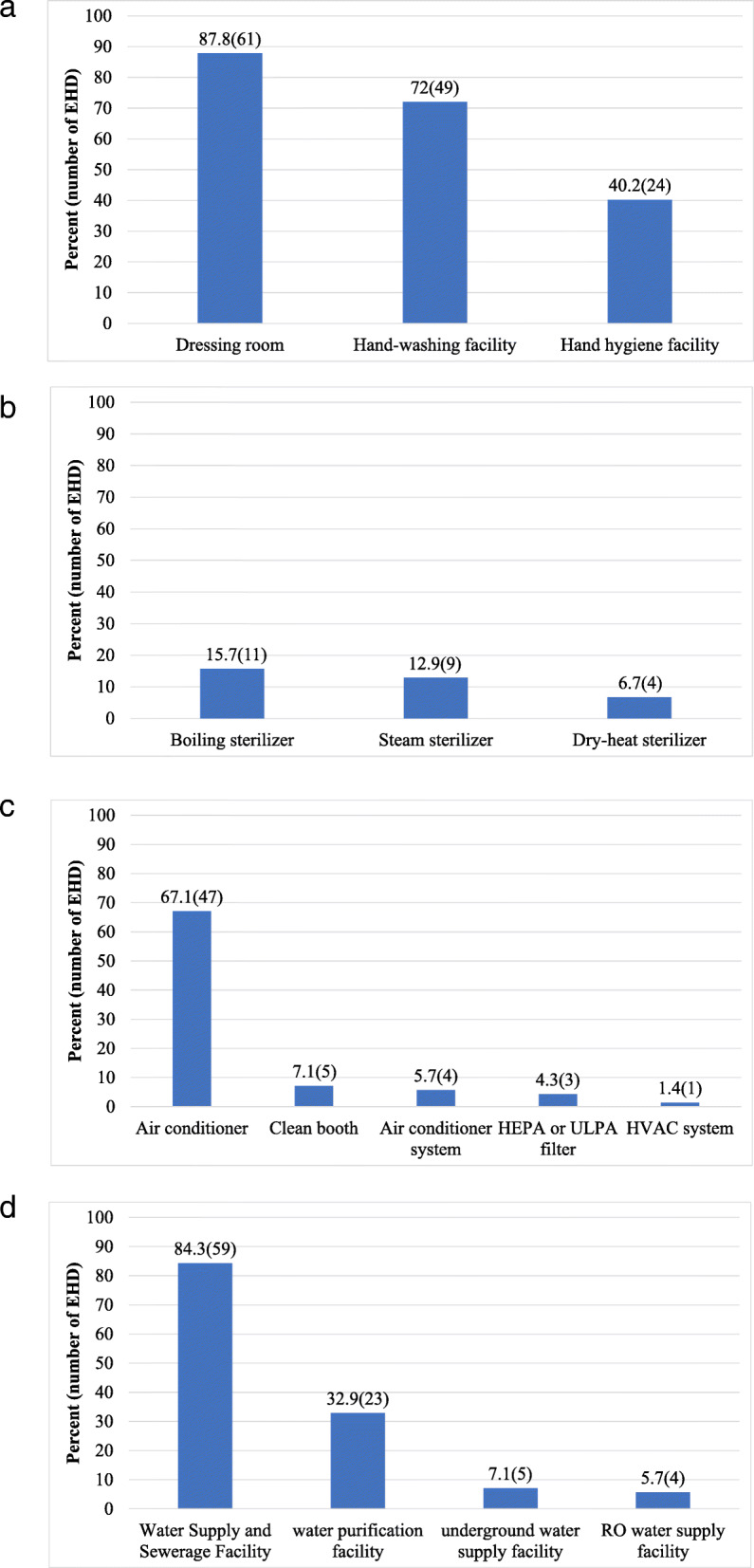
Facility status in herbal medicine-external herbal dispensary (H-EHD). HEPA: High Efficiency Particulate Air; HVAC: Heating, Ventilation, & Air Conditioning; RO: Reverse Osmosis; ULPA: Ultra-Low Particulate Air

Regarding hygiene management facility, 87.8% (*n* = 61) of the H-EHDs had a dressing room, while 72.0% (*n* = 49) had a hand-washing room. 40.2% (*n* = 24) had a hand disinfection facility (Fig. 2(a)).

Regarding pest control facility, 44.3% (*n* = 31) of the H-EHDs had pest control lamps, 40.0% (*n* = 28) had pest traps, and 27.1% (*n* = 19) had ultrasonic devices.

81.4% (*n* = 57) of H-EHDs had an extraction equipment, 41.4% (*n* = 29) had a distiller, and 41.4% (*n* = 29) had an evaporator.

With regards to sterilization facilities, 15.7% (*n* = 11) of H-EHDs had a boiling sterilizer, 12.9% (*n* = 9) a steam sterilizer, and 6.7% (*n* = 4) a dry-heat sterilizer (Fig. 2(b)).

57.1% (*n* = 40) of H-EHDs had automatic filling equipment, while 17.1% (*n* = 12) had semi-automatic filling equipment. 8.6% (*n* = 5) had manual filling equipment.

21.4% (*n* = 15) of H-EHDs had a manual foreign body inspection facility, 1.4% (*n* = 1) had a semi-automatic foreign body inspection facility, and 1.4% (*n* = 1) had an automatic foreign body inspection facility.

90.0% (*n* = 63) of H-EHDs had a raw material warehouse, 47.1% (*n* = 33) a final product warehouse, and 25.7% (*n* = 18) had a semi-finished product warehouse.

With regards to their cleanliness management facilities, 67.1% (*n* = 47) of H-EHDs had an air-conditioner, 7.1% (*n* = 5) had clean booths, 5.7% (*n* = 4) had air conditioner systems, 4.3% (n = 3) had HEPA or ULPA filters, and 1.4% (*n* = 1) had HVAC systems (Fig. 2(c)).

None of the H-EHDs had facilities to prevent cross-contamination.

Concerning their water supply facilities, 84.3% (*n* = 59) of H-EHDs had a water supply facility, 32.9% (*n* = 23) had purified water supply facility, 7.1% (*n* = 5) had underground water supply facility, and 5.7% (*n* = 4) had RO water supply facility (Fig. 2(d)).

### Quality control status of pharmacopuncture-external herbal dispensaries (P-EHD)

The monitoring frequency of temperature and humidity in the storage facilities for raw materials and final products in P-EHDs was as follows: 36.4% (*n* = 4) monitored every day, 36.4% (*n* = 4) once a week, and 27.2% (*n* = 3) once a month.

As for the management of poisonous herbs, 54.5% (*n* = 6) of P-EHDs used a TKM pharmacist to manage poisonous herbs, 45.5% (n = 5) stored them separately from other medicinal herbs, and 9.1% (*n* = 1) had no management systems in place.

**Fig. 3:**
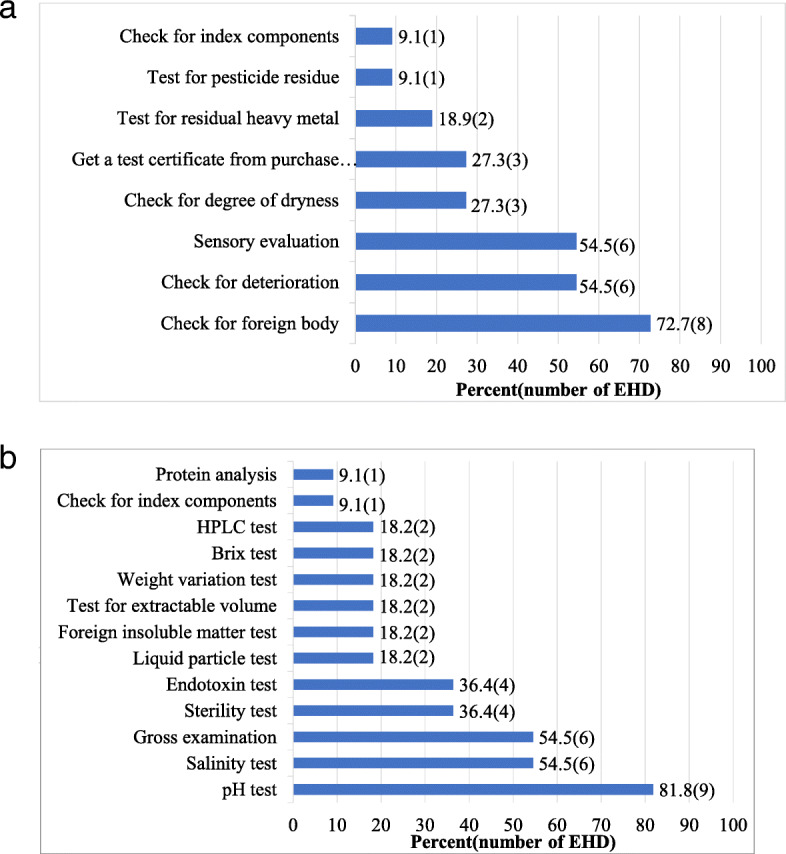
Quality control status in pharmacopuncture-external herbal dispensary (P-EHD). HPLC: High-Performance Liquid Chromatography

With regard to the management of the quality of non-medicinal herbs, the P-EHDs were found to, check for foreign body (72.7%), check for deterioration (54.5%), conduct sensory evaluations (54.5%), check for degree of dryness (27.3%), obtain certification from purchase place (27.3%), test for residual heavy metal (18.9%), test for pesticide residue (9.1%), and check for index components (9.1%) (Fig. 3(a)).

The quality control measures implemented by pH test (81.8%), salinity test (54.5%), gross examination (54.5%), sterility test (36.4%), endotoxin test (36.4%), liquid particle test (18.2%), foreign insoluble matter test (18.2%), test for extractable volume (18.2%), weight variation test (18.2%), brix test (18.2%), High-Performance Liquid Chromatography (HPLC) test (18.2%), check for index components (9.1%), and protein analysis (9.1%) (Fig. 3(b)).

### Quality control status of herbal medicine-external herbal dispensaries (H-EHD)

The monitoring frequency of temperature and humidity in the storage facilities for raw materials and finish products in the H-EHDs was as follows: 22.9% (*n* = 16) monitored every day, 36.4% (*n* = 27) once a week, 30.0% (*n* = 21) once a month, and 8.6% (*n* = 6) twice a month.

As for the management of poisonous herbs, 72.9% (*n* = 51) of H-EHDs used a TKM pharmacist to manage poisonous herbs, 41.4% (*n* = 29) stored them separately from other medicinal herbs and 5.7% (*n* = 4) had no management systems in place.

**Fig. 4:**
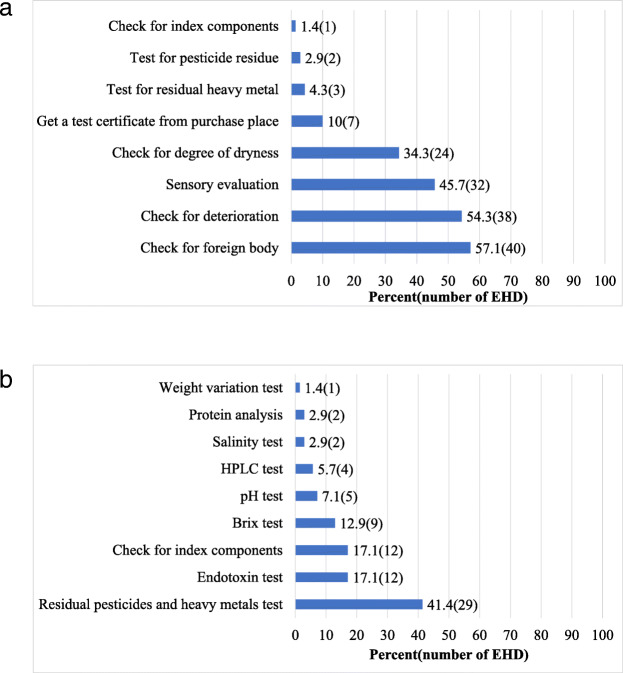
Quality control status in herbal medicine-external herbal dispensary (H-EHD). HPLC: High-Performance Liquid Chromatography

With regard to the management of the quality of non-medicinal herbs, the H-EHDs were found to, check for foreign body (57.1%), check for deterioration (54.3%), conduct sensory evaluation (45.7%), check for degree of dryness (34.3%), obtain certification from purchase place (10.0%), test for residual heavy metal (4.3%) test for pesticide residue (2.9%), and check for index components (1.4%) (Fig. 4(a)).

The quality control measures implemented by H-EHDs for HMs (final products) included a residual pesticides and heavy metals test (41.4%), check for index components (17.1%), endotoxin test (17.1%), brix test (12.9%), pH test (7.1%), HPLC test (5.7%), salinity test (2.9%), protein analysis (2.9%), and weight variation test (1.4%) (Fig. 4(b)).

### Comparison of drug management statuses of KGMP, legal requirements of EHD and survey items

Table 3 shows the comparison of drug management of KGMP, legal requirements of EHD and survey items. KGMP is composed of 10 categories and 38 items, and the legalized area related to EHD standards correspond to 3 categories and 7 items. In the end, 6 items, excluding 1 item relating to TKM pharmacists, out of the 7 EHD criteria met the KGMP criteria.

**Table 3:** Comparison on drug management of KGMP, EHD and survey item

Category	KGMP	Legal requirements of EHD	Survey item
**Facilities and equipments**	* water supply facility	* water supply facility	* water supply facility
	* manufacturing facility and equipment	* manufacturing facility and equipment	* manufacturing facility and equipment
			- Extraction, evaporation, or distillation
			- Filling facilities
	* warehouse (raw herbs, semi-finished products, final products)	* warehouse (raw herbs, final products)	* warehouse (raw herbs, semi-finished products, final products)
	* pest control facility	* pest control facility	* pest control facility
	* hygiene management facility	* hygiene management facility	* hygiene management facility
	* sterilization facility		* sterilization facility
	* Inspection facility		* Inspection facility
	* cleanliness management facilities		* cleanliness management facilities
	* cross-contamination prevention facility		* cross-contamination prevention facility
	* drainage facility		
	* lighting		
**Organization and personnel**	* operation of quality control unit * initial and continuing training	* arrangement of a TKM pharmacist	
**Documentation** **(generation, control, retention)**	* drug master files * quality control standard document * manufacturing control standard document * hygiene control standard document		
**Validation**	* process * testing method * cleaning * utility system * computer system * packing		
**Manufacture hygienic management**	* sanitation and hygiene of personnel * hygienic management of production area * cleaning of manufacturing equipment	* cleaning of preparing equipment	
**Quality control**	* monitoring of temperature and humidity * stability testing * product quality review		* monitoring of temperature and humidity
			* poisonous herbs control
			* quality control
			- testing raw herb
			- testing herbal medicine (finished product)
**Production and process controls**	* production process control * packing process control * product return and re-packing		
**Warehousing and distribution procedures**	* warehousing control * storage control * distribution control		
**Complaints and product recall**	* response and measure to customer complaint * product recall and measure		
**Self inspection**	* independent inspection by internal personnel and external institution		

*EHD* external herbal dispensary, *KGMP* Korea good manufacture practice, *TKM* traditional Korea medicine

## Discussion

As this is the first government-driven full-scale survey to examine the current status of EHD, 81 EHDs (completion rate: 75.7%) were surveyed. In this manner, current data to provide detailed facility and equipment criteria with regard to the five facility criteria in the existing Medical Act was secured. The EHDs surveyed in this study provide TKM clinics with PHMs and play an important role in providing primary traditional medical care [5, 27]. To inform the policies that determine the management criteria for the EHDs, which are one of two sources of HMs (namely, those from pharmaceutical companies and those prepared in EHDs), the researchers gathered in-depth information on the facilities and quality control of EHDs and would like to make some recommendations to improve policies related to EHDs.

EHDs in South Korea are divided into two types: those that dispense PHMs (including the decoction types, pills, powders, liquids, etc.) and pharmacopunctures, which have been developed since the 1970s and used for pain control as well as gynecological and musculoskeletal diseases [24, 28]. Pharmacopuncture is regarded as one of the most commonly used treatment methods in TKM institutions and is now covered by car insurance in South Korea [28]. More specifically, pharmacopuncture is a treatment method where PHMs are injected into acupunctural points for treating various types of diseases. This resembles typical injections in the medical setting and the preparation process is different from those of PHMs [28]. As separate facility management criteria are required to inject pharmacopunctures and PHMs that are orally administered, the researchers conducted separate analyses to account for the different level of quality control of those two categories.

The results showed that 90.9% of P-EHDs had self-quality inspection facilities. As pharmacopuncture involves injection of the liquid-form medicine into the body, sterility in production must be maintained. It is also believed that P-EHDs are endeavoring to ensure safety by providing test reports to the TKM practitioners after self-inspecting their pharmacopuncture medicinal fluids. However, only 7.1% of H-EHDs had self-inspection facilities, and 37.2% of the H-EHDs did not perform any inspection of PHMs. Moreover, HMs have been used to treat illness across the world for thousands of years, and is used widely by Koreans today. Therefore, as an empirical medical practice, TM is considered to be safe, and the awareness on the need to verify the safety of pharmacopunctures was found to be rather low [25, 29]. However, it is necessary that the verification of TM be provided to the public, so that TM can serve as a pillar of primary care in our healthcare system. Therefore, we recommend EHDs obtain self-inspection facilities to conduct self-safety inspections.

It was also found that less than 50% of both H-EHDs and P-EHDs were equipped with pest-control equipment, such as insect catcher lamps or ultrasonic pest traps. Pest control is one of the key sanitary operational practices of the GMP and is a preventive measure guarding against contamination of raw materials and medicines [28, 29]. Therefore, to prevent contamination of herbal materials and prepared PHMs, it is necessary that EHDs be equipped with pest control facilities.

In the case of P-EHDs, 100% had steam sterilizers, and 71.7% had dry-heat sterilizers. As for the H-EHDs, no more than 15% had any of these. Therefore, it can be said that most of the PHMs prepared in H-EHDs do not go through sterilization processes. In this context, GMP guidelines recommend the provision of sterilization facilities in the preparation process and the recording of the sterilization processes (e.g. used equipment, data, time, product name and lot number of each batch processed in the equipment) [30]. While such prepared PHMs are not licensed by the MFDS, it would be necessary to introduce sterilization as per the GMP guidelines in order to ensure that the medication is safe.

As for the P-EHDs, they had the equipment necessary for cleanliness management. However, only some of them had air-filtration systems (63.6% had air-conditioners, 81.8% had HEPA or ULPA filters, and 27.3% had HVAC systems). As for H-EHDs, 67.1% had air-conditioners but most did not have proper filtration systems. According to the laws on the management of medicines of the Food & Drug Agency (FDA), USA, the production areas of medicines are required to have air filtration systems and equipment to ensure proper air supply [31]. The air filtration systems and equipment are required to control the air pressure, micro-organisms, dust, humidity, and temperature in all processes of production, packaging, and storage of medicines [31]. While it would be desirable to have cleanliness management systems to ensure the safety of medicines, it is believed that EHDs will find it difficult to invest in such systems due to the lack of funds, as they supply some 15,000 TKM clinics in South Korea with PHMs. In the future, the management criteria of EHDs would have to introduce cleanliness management systems following feedback from the field and to safeguard public health safety.

Moreover, 84.3% of H-EHDs were found to be equipped with a water supply and 32.9% with purified water supply systems, while 90.9% of P-EHDs were equipped with a water supply, 72.7% with purified water supply systems, 54.5% with ultrapure water systems, and 45.5% with injection water systems. According to GMP guidelines, the injection fluids, which are produced using injection water, must be sterile [32, 33]. To ensure sterility of the pharmacopuncture fluids, the criteria for water use must be provided. This must be managed in accordance with the water criteria for sterile products in the GMP guidelines.

As for the management of poisonous herbs, these were being managed by the TKM pharmacists themselves in more than 50% of EHDs, while more than 40% of them had them separated from ordinary herbs. The MFDS has designated 21 poisonous herbs, which are off-limits for purchase for non-licensed persons and are supplied only to TKM clinics [34]. Poisonous herbs may cause severe side effects or even death if taken above a certain level [35]. Thus, they need to be managed separated from normal herbal materials by licensed TKM pharmacists. If a non-licensed employee mistakenly adds toxic herbs to the preparation process of PHMs, this can be a matter of life and death for a patient. Therefore, it is important that the safety criteria for toxic herbs is clearly established.

Quality control for non-medicinal herbs was focused on checking for foreign bodies and deterioration of the herbs along with sensory evaluation [31]. According to the Korean Medical Act, TKM clinics, including EHDs, must only use the 601 medicinal herbs approved by MFDS in South Korea [36]. Meanwhile, the non-medicinal herbs—which are not included in the list of 601 approved herbs but are still used in EHDs after going through in-depth inspections, foreign body inspections, or quality inspections—do not undergo tests for residual heavy metals or pesticide residues. In the future, safety control test criteria would have to be established, and all medicinal herbs must undergo tests for heavy metals, pesticides, aflatoxins, sulfur dioxide, and benzopyrene. Only those that passed these tests should be used as safe herbal medicinal materials for TM users.

### Patient safety and revision of EHD legal standards

Currently, the facility standards for EHD stipulates that 5 facilities (water supply, manufacture, pest control facility, hygiene management, and warehousing) are legally necessary. However, if there is no sterilization facility and sterilization of the final products cannot be achieved, there is a possibility that microbial life may occur, causing the herbal medicine to deteriorate. This could adversely affect the human body when herbal medicine is ingested [37, 38]. Moreover, if an air conditioner is installed in the EHD and the temperature and humidity suitable for the HM are not managed, the HM may deteriorate. Additionally, since toxic herbs can be mixed in the process of preparing HMs, if a person with HM management qualifications is not responsible and managed, fatal side effects may occur to patients.

The aim of GMP guidelines is to assist the development and implementation of effective quality risk management, covering activities such as research and development, sourcing of materials, manufacturing, packaging, testing, storage and distribution [39]. However, when quality control measures are less effective, patients may be put at risk through the production of medicines of inadequate standards [39]. As such, standards for facility and quality control are directly related to patient safety, and legal standards for EHD need to be revised by referring to the survey results. Besides, since the Medical Act comprehensively stipulates the EHD facility standards without sufficient details (facility or device type), it is necessary to present the details of the facility standards based on the survey results.

### Study limitations

The limitations of this study were as follows. First, the questionnaires were developed through meetings between the researchers and the external experts. Further, reviews were conducted by them in order to understand the current status of the facilities and quality control processes. However, the reliability and validity of this questionnaire was not confirmed. Nevertheless, if the reliability and validity of the study is to be confirmed via the questionnaires, it would be possible to understand the current status of the EHDs more accurately. Second, as the current investigation is based on a self-reported questionnaire, the results are dependent on the responses of the participants. For an accurate understanding of the current status of the facilities and quality control processes, an expert and a researcher would have to manually verify the same. Third, we provided small gifts to the participants in order to ease the process of securing the interviewees, and this may cause a bias that guided the interviewees to provide more positive answers. Lastly, survey items were limited to 2 areas of facility and quality control, and survey items were not included in the other areas (e.g. organization, documentation, and validation) suggested by the GMP guidelines. To strengthen the EHD standards in a step-by-step process, it is essential to conduct an accurate current status survey on the operational status of each area, and based on this, the revisions to the standards must then be made.

### Future challenges

Currently, the manufacturing criteria for medicines are internationally harmonized in accordance with the guidelines of the International Council of Harmonization (ICH). In the case of traditional herbal products, the harmonization of the control criteria in the Asian regions was in accordance with the Association of South East Asian Nations (ASEAN) guidelines [40, 41]. As per the ASEAN guidelines for traditional herbal products, quality control is concerned with sampling, specifications, testing, organization, documentation, and release procedures that ensure that the necessary tests are in fact carried out [41]. However, while the quality control of EHDs included sampling, specifications, and testing to a certain degree, there was no mention of the organization, documentation, and release procedures. It is worth noting that the international guidelines are applicable to the herbal products manufactured by pharmaceutical companies, while the PHMs prepared in TM clinics or EHDs follow these guidelines without being required to do so by the law. This can cause difficulties in implementation of guidelines to improve the quality control of EHDs in the Korean context.

Moreover, the future challenges for securing safety and quality management of PHMs include the following: (1) investigation of EHDs’ operation statuses in the areas not included in this study (e.g. organization, documentation, and validation) as suggested by GMP guidelines; (2) the establishment of mandatory criteria by government, including the legal basis; (3) and the international harmonization of EHDs by the creation of a discussion body with the countries in which TMs are practiced. In addition, it is necessary to provide a specific set of quality management criteria through a comparative study of the international guidelines for TMs (e.g. International Council of Harmonization guidelines, Association of Southeast Asian Nations guidelines) for the H-EHDs, where the HMs that are orally administered are prepared, and P-EHDs, where the herbal acupunctures for injections are prepared.

## Conclusions

Based on the results of this study, 6 EHD legal standard items out of 38 KGMP criteria were met. This study investigated detailed facilities, equipment, and quality control methods for each EHD facility and quality control item, and established the basis for revising the legal standards of EHD. In the future, it is necessary to revise EHD standards or introduce an evaluation system to ensure safe and reliable HM for patients.

## Supplementary information

**Additional file 1.** Current status of external herbal dispensaries where preparing traditional herbal medicines in South Korea: A survey. The final questionnaire used for collection of data.

## Data Availability

The datasets used and/or analyzed during the current study are available from the corresponding author upon reasonable request.

## Abbreviations

EHD: External herbal dispensary
EO: Ethylene oxide
FDA: Food & Drug Agency
GMP: Good manufacture practice
H-EHD: Herbal medicine-external herbal dispensary
HEPA: High efficiency particulate air
HMs: Herbal medicines
HVAC: Heating, ventilation, & air conditioning
KAHAS: Korea Agency of Hazard analysis and critical control point Accreditation and Services
MFDS: Ministry of Food and Drug Safety
MoHW: Ministry of Health and Welfare
P-EHD: Pharmacopuncture-external herbal dispensary
PHMs: Personalized herbal medicines
RO: Reverse osmosis
TKM: Traditional Korean medicine
TM: Traditional medicine
ULPA: Ultra-low particulate air
