# Supplementary material for: Current status and future perspective of external herbal dispensaries preparing traditional herbal medicine in South Korea: the first National-Wide Survey results
Source: BMC Complement Med Ther. 2020 Nov 23;20:354. doi: 10.1186/s12906-020-03094-0 (PMC7681953; doi:10.1186/s12906-020-03094-0)
Supplement: Supplementary file 1 — Additional file 1. Current status of external herbal dispensaries where preparing traditional herbal medicines in South Korea: A survey. The final questionnaire used for collection of data. [file 12906_2020_3094_MOESM1_ESM.docx]

# Current Status of External Herbal Dispensaries Preparing Traditional Herbal Medicines in South Korea: A Survey

| **Managing Department** | **Agent in Charge of the Survey** |
| --- | --- |
|  | **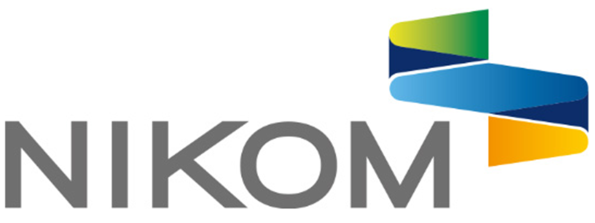** |
| **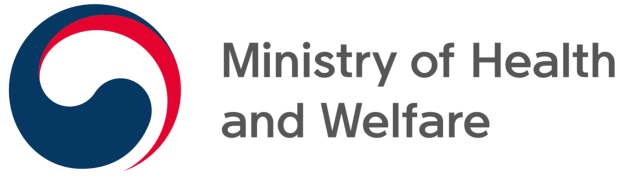** |  |

**[Objectives]**

By collecting data on the current status of the facilities and quality control of External Herbal Dispensaries (EHD) of traditional medicine (TM) institutions, we hope to

1. **Provided evidence for the establishment of criteria on the management of facilities and quality control of EHDs.**
2. **Establish quality control systems for the concocted herbal medicines (or pharmacopuncture) from EHDs.**
3. **Investigate the differences between herbal EHDs (H-EHDs) and pharmacopuncture EHDs (P-EHDs).**

**[Targets]**

All EHDs in South Korea as of 2017.

**[Items]**

The survey items comprised three parts:

1. **Basic status: type of EHD; location; opening year; total area; type of medical institution.**
2. **Facility status: self-quality inspections; hygiene management; pest control; equipment for extraction, evaporation, or distillation; sterilization; foreign body inspection; storage for herbs, semi-finished, or final products; cleanliness management; cross-contamination prevention; water supply.**
3. **Quality control status: the frequency of monitoring temperature and humidity; quality control of poisonous herbs; quality control of final products.**

**[Instructions]**

The Ministry of Health and Welfare intends to conduct a survey to examine the current status of facilities and quality control of the EHDs of TM institutions that prepare personalized herbal medicines (PHMs) or pharmacopunctures. Your answers will contribute to the establishment of criteria for the management of facilities and quality control in EHDs. The contents of this questionnaire will be used in statistical analysis for academic purposes only, and any personal information will remain strictly confidential other than for statistical analyses.

Please read and observe the following instructions as you complete the questionnaire;

1. **This questionnaire must be completed by the head of the institution or the person in your institution who is responsible for the preparation of PHMs (a TM pharmacist) and well informed on the current status of facilities and quality control practices.**
2. **While the majority of questions are multiple-choice, some require you to give short responses.**
3. **All responses must reflect the current status as of the time of your completing of this questionnaire.**
4. **If you cannot find the option that best describes your response, you can fill in the blanks with relevant information on the facilities or quality control practices in (other _______).**

**[PART 1. Demographic information]**

**1. Under what category is your affiliated external herbal dispensary classified?**

① External herbal dispensary where preparing pharmacopuncture

② External herbal dispensary where preparing herbal medicine

**2. Where is your affiliated external herbal dispensary located?**

| ① Seoul ② Busan ③ Incheon ④ Daegu ⑤ Gwangju ⑥ Daejeon ⑦ Ulsan ⑧ Sejong  ⑨ Gyunggi ⑩ Gangwon ⑪ Chungbuk ⑫ Chungnam ⑬ Jeonbuk ⑭ Jeonnam  ⑮ Gyeongbuk ⑯ Gyeongnam ⑰ Jeju |
| --- |

**3. In what year was your EHD established?**

* The EDH system has been in place since 2009.

① 2009 ② 2010 – 2012 ③ 2013 – 2015 ④ 2016 – 2017

**4. How wide is your EHD?:** (_______________m^2^)

**5. What category does the TM institution with the EHD belong to?**

① Traditional Korean medicine clinics (primary healthcare institution)

1. ② Network Traditional Korean medicine clinics (primary healthcare institution)
2. ③ Traditional Korean medicine hospital (Secondary healthcare institution)
3. ④ Public health center
4. ⑤ Other: _______________

**[PART 2. Facility status]**

**1. Do you have the facilities for self-quality inspections?**

(1) We have one. (2) We use an accredited institution for this purpose. (3) We have the pharmaceutical companies do the inspections for us. (4) Other ________

**2. What type of hygiene management facilities do you have in place?**

| **Hygiene management facilities** | |
| --- | --- |
| A. Dressing room | Yes □ No □ |
| B. Hand-washing room | Yes □ No □ |
| C. Hand disinfection facility | Yes □ No □ |
| D. Other:________________ | Yes □ No □ |

**3. What kind of pest control facilities do you have in place?**

| **Pest control facilities** | |
| --- | --- |
| A. Ultrasonic device | Yes □ No □ |
| B. Pest control lamp | Yes □ No □ |
| C. Pest trap | Yes □ No □ |
| D. Other:________________ | Yes □ No □ |

**4. What kind of extraction, evaporation, or distillation equipments do you have?**

| **Extraction, evaporation, or distillation equipments** | |
| --- | --- |
| A. Extraction equipment | Yes □ No □ |
| B. Distillation equipment | Yes □ No □ |
| C. Evaporation equipment | Yes □ No □ |

**5. What kind of sterilization facilities do you have?**

| **Sterilization facilities** | |
| --- | --- |
| A. Steam sterilizer | Yes □ No □ |
| B. Dry-heat sterilizer | Yes □ No □ |
| C. EO gas sterilizer | Yes □ No □ |
| D. Boiling sterilizer | Yes □ No □ |
| E. Other:________________ | Yes □ No □ |

* EO: Ethylene Oxide

**6. What kind of filling facilities do you have?**

| **Filling facilities** | |
| --- | --- |
| A. Automatic filling equipment | Yes □ No □ |
| B. Semi-automatic filling equipment | Yes □ No □ |
| C. Manual filling equipment | Yes □ No □ |
| D. Other:________________ | Yes □ No □ |

**7. What kind of foreign body inspection facilities do you have in place?**

| **Foreign body inspection facilities** | |
| --- | --- |
| A. Automatic foreign body inspection facility | Yes □ No □ |
| B. Semi-automatic foreign body inspection facility | Yes □ No □ |
| C. Manual foreign body inspection facility | Yes □ No □ |
| D. Other:________________ | Yes □ No □ |

**8. What kind of raw materials, semi-finished products, or final products storage facilities do you have?**

| **Warehouse of raw materials, semi-finished products, or final products** | |
| --- | --- |
| A. Raw material warehouse | Yes □ No □ |
| B. Semi-finished product warehouse | Yes □ No □ |
| C. Final product warehouse | Yes □ No □ |

**9. What kind of cleanliness management facilities do you have?**

| **Cleanliness management facilities** | |
| --- | --- |
| A. Clean booth | Yes □ No □ |
| B. Air conditioner | Yes □ No □ |
| C. Air conditioner system | Yes □ No □ |
| D. HEPA or ULPA filter | Yes □ No □ |
| E. HVAC system | Yes □ No □ |
| F. Other:________________ | Yes □ No □ |

* HEPA: High Efficiency Particulate Air; HVAC: Heating, Ventilation, & Air Conditioning;

ULPA: Ultra-Low Particulate Air

**10. What kind of cross-contamination prevention facilities do you have in place?**

| **Cross-contamination Prevention facilities** | |
| --- | --- |
| A. Air shower room | Yes □ No □ |
| B. Pass through box | Yes □ No □ |
| C. Door interlock facility | Yes □ No □ |
| D. Other:________________ | Yes □ No □ |

**11. What kind of water supply facilities do you have?**

| **Water supply facilities** | |
| --- | --- |
| A. Water supply and sewerage facility | Yes □ No □ |
| B. Underground water supply facility | Yes □ No □ |
| C. RO water supply facility | Yes □ No □ |
| D. Water purification facility | Yes □ No □ |
| E. Water for injection supply facility | Yes □ No □ |
| F. Deionized water supply facility | Yes □ No □ |
| G. Sterile purified water supply facility | Yes □ No □ |
| H. Other:________________ | Yes □ No □ |

* RO: Reverse Osmosis

**[PART 3. Quality control status]**

**1. How often do you monitor the temperature and humidity in the storage for the raw materials and final products?**

**(_____________) time(s) a month**

**2. How are you managing your poisonous herbs? (Select all the answers that apply to you.)**

* Poisonous herbs refer to the following 21 medicinal herbs specified by law by the Ministry of Food and Drug Safety (MFDS); Euphorbiae Kansui Radix (甘遂), Calomelas (輕粉), Euphorbiae Fischerianae Radix (狼毒), Lithargyrum (密陀僧), Cantharides (斑猫), Pinelliae Tuber (半夏), Aconiti Koreani Tuber (白附子), Strychni Ignatii Semen (寶豆), Aconiti Lateralis Radix Preparata (附子), Bufonis Venenum (蟾酥), Euphorbiae Lathyridis Semen (續隨子), Hydrargyrum (水銀), Lini Semen (亞麻仁), 鉛丹 (Minium), Realgar (雄黃), Cinnabaris (朱砂), Arisaematis Rhizoma (天南星), Aconiti Tuber (川烏), Aconiti Kusnezoffii Tuber (草烏), Crotonis Semen (巴豆), Nux Vomica (馬錢子)

(1) Store separately from medicinal herbs (2) Managed by traditional medicine pharmacist

(3) Have no management system (4) Other_____________

**3. How to you manage the quality of the non-medicinal herbs?**

* A non-medicinal herbs are a raw herbs that have not been manufactured in an herb-GMP company approved by the ministry of food and drug safety (MFDS).

| **Quality control of non-medical herbs** | |
| --- | --- |
| A. Check for foreign body | Yes □ No □ |
| B. Check for deterioration | Yes □ No □ |
| C. Sensory evaluation | Yes □ No □ |
| D. Check for degree of dryness | Yes □ No □ |
| E. Get a certificate from purchase place | Yes □ No □ |
| F. Test for residual heavy metal | Yes □ No □ |
| G. Test for pesticide residue | Yes □ No □ |
| H. Check for index components | Yes □ No □ |
| I. Other:________________ | Yes □ No □ |

**4. How to you do the quality control of the finished products (personalized herbal medicines or pharmacopuncture)?**

| **Quality control of final product** | |
| --- | --- |
| A. pH test | Yes □ No □ |
| B. Salinity test | Yes □ No □ |
| C. Gross examination | Yes □ No □ |
| D. Sterility test | Yes □ No □ |
| E. Endotoxin test | Yes □ No □ |
| F. Liquid particle test | Yes □ No □ |
| G. Foreign insoluble matter test | Yes □ No □ |
| H. Test for extractable volume | Yes □ No □ |
| I. Weight variation test | Yes □ No □ |
| J. Brix test | Yes □ No □ |
| K. HPLC test | Yes □ No □ |
| L. Check for index components | Yes □ No □ |
| M. Residual pesticides and heavy metals test | Yes □ No □ |
| N. Other:________________ | Yes □ No □ |

* HPLC: High-Performance Liquid Chromatography
